# Meditation and Yoga for Irritable Bowel Syndrome: A Randomized Clinical Trial

**DOI:** 10.14309/ajg.0000000000002052

**Published:** 2022-10-11

**Authors:** Adrijana D'Silva, Deborah A. Marshall, Jeff K. Vallance, Yasmin Nasser, Vidya Rajagopalan, Jessie H. Szostakiwskyj, Maitreyi Raman

**Affiliations:** 1 Department of Community Health Sciences, Cumming School of Medicine, University of Calgary, Calgary, Canada;; 2 Arthur J.E. Child Chair, Department of Medicine, Cumming School of Medicine, University of Calgary, Calgary, Canada;; 3 Faculty of Health Disciplines, Athabasca University, Athabasca, Canada;; 4 Division of Gastroenterology and Hepatology, Department of Medicine, Cumming School of Medicine, University of Calgary, Calgary, Canada;; 5 Snyder Institute for Chronic Diseases, Cumming School of Medicine, University of Calgary, Calgary, Canada;; 6 Clinical Research Unit, Cumming School of Medicine, University of Calgary, Calgary, Canada.

## Abstract

Delivered in person, yoga is effective in managing irritable bowel syndrome (IBS) symptoms. The evidence for efficacy, feasibility, and safety of virtually delivered yoga for patients with IBS is unknown.

**METHODS:**

Adults diagnosed with IBS were randomized to either Hatha yoga intervention of 8 weekly online classes delivered virtually or an advice-only control group and assessed at baseline and postintervention. We used an unadjusted ANOVA to determine differences between and within groups on the primary outcome (decrease of ≥50 points in IBS Symptom Severity Scale [IBS-SSS]) and secondary outcomes (quality of life, anxiety and depression, fatigue, somatic symptoms, perceived stress, COVID-19 stress, and self-compassion). We assessed feasibility through recruitment and attrition rates, adherence, participant satisfaction, and safety (i.e., adverse events).

**RESULTS:**

Seventy-nine people participated (mean age 45.4 years [SD = 14.0], 92% women, 20% attrition rate). IBS-SSS decreased significantly in the treatment group (Δ_change_ = 54.7, *P* = 0.028), but not in the control group (Δ_change_ = 22.6, *P* = 0.277). Fourteen patients (37%) in the yoga group reached a clinically relevant decrease of ≥50 points on the IBS-SSS postintervention compared with 8 patients (20%) in the control group (*P* = 0.242). No significant difference was found between groups in IBS-SSS score postintervention (*P* = 0.149), but significant differences in favor of the treatment group for quality of life (*P* = 0.030), fatigue (*P* = 0.035), and perceived stress (*P* = 0.040) were identified. The yoga program demonstrated feasibility. Intention to practice yoga decreased significantly in both groups from baseline to postintervention (*P* < 0.001). However, the decline in intention did not correlate with practice minutes.

**DISCUSSION:**

Virtually delivered yoga is safe and feasible, and effective in reducing IBS symptoms. Based on the primary end point, the intervention was not superior to an advice-only control group.

## INTRODUCTION

Irritable bowel syndrome (IBS) is a common chronic condition frequently involving alterations of the gut-brain axis. IBS is associated with psychiatric comorbidities, incomplete symptom control, and impaired quality of life (QOL) (1). Altered stress response from psychological and physiological mechanisms may contribute to altered brain-gut signaling patterns and IBS symptoms (2). Therapies focusing on mind-body interactions and stress reduction may be adjunctive treatments for IBS.

Yoga is a mind-body therapy that includes physical postures (asanas), breathing exercises (pranayama), and meditation (3) to improve physiological, psychological, and emotional health (4). In-person yoga interventions have been evaluated for IBS in adult and adolescent populations (5–12), demonstrating feasibility and effectiveness in reducing IBS symptoms while also improving QOL and mental health. Proposed mechanisms of action for yoga target the brain-gut axis directly by reducing sympathetic activity, increasing parasympathetic activity, and modulating hypothalamus-pituitary-adrenal axis function (13). Yoga may be as effective as pharmacotherapy, cognitive-behavioral therapy, exercise, and the low FODMAP diet to reduce IBS symptoms, symptoms of anxiety and depression, and stress in patients with IBS (3,14,15). The rationale for virtually delivered interventions to manage IBS is increasing due to limited healthcare resources and higher cost-effectiveness (16,17). A yoga program delivered virtually is convenient, feasible, and effective in a COVID-19 setting in other diseases (e.g., cancer, heart disease, or chronic obstructive pulmonary disease) (18–20) and postpandemic where virtual care is accepted and expected. Effectiveness outcomes from these intervention nonrandomized studies included improved symptoms and functional performance, QOL, sleep quality, mental health, and reduced fatigue. To date, no studies have yet explored demonstrate the feasibility and efficacy of virtually delivered yoga for patients with IBS.

The primary objective of the Meditation and Yoga for Irritable Bowel Syndrome (MY-IBS) study was to examine the efficacy and feasibility of a virtual 8-week yoga program on IBS symptom severity compared with an advice-only control group. Secondary objectives were to determine (i) whether a virtual yoga program improves the QOL, mental health outcomes, perceived stress, fatigue, COVID-19–related stress, and self-compassion, and (ii) the level of intention to practice yoga at baseline and whether intention correlated with practice minutes.

## METHODS

### Study design overview

MY-IBS was a randomized 2-group controlled trial conducted at the University of Calgary in Alberta, Canada, from March 2021 to December 2022. Participants were not blinded to trial arms. Eligible participants diagnosed with IBS based on Rome IV (21) criteria were referred to the study by a healthcare professional (e.g., physician, nurse, or dietician), were aged 18–70 years, had an adequate understanding of English, scored at least 75 of 500 points on the IBS Symptoms Severity Scale indicating at least mild IBS symptoms (22), and were on stable doses of medications for IBS (including antidepressants) without major changes to diet or physical activity patterns for at least 8 weeks before starting the intervention. Participants were permitted to continue with their current therapies during the study period, and no new medications were permitted during the trial period. Exclusion criteria included a major physical impairment that would prevent the individual from doing yoga determined by either the patient or the study coordinator and diagnosis of any major cognitive, psychological, or psychiatric disorder (e.g., major depression or schizophrenia) as identified by the treating physician or healthcare practitioner or screened by the study coordinator using the Patient Health Questionnaire-9 (PHQ-9). Individuals who scored 20 points or higher on the PHQ-9 indicating severe depression were not eligible to participate.

Individuals across Canada were eligible to participate. Participants were recruited between March and October 2021 and identified through (i) gastroenterology clinics across Calgary, Alberta, (ii) a previous survey where participants indicated an interest in this study and provided consent to be contacted, (iii) social media, (iv) self-referrals, (v) Canadian Association of Gastroenterologists monthly newsletter, and (vi) the IMAGINE (Inflammation, Microbiome and Alimentation Gastrointestinal and Neuropsychiatric Effects) cohort study at the University of Calgary. The University of Calgary Conjoint Health Research Ethics Board approved this study (ID: REB20-0084).

### Interventions

#### Yoga intervention group.

The details of the yoga program have been published elsewhere (23) (see Supplementary Table 1, Supplementary Digital Content 1, http://links.lww.com/AJG/C766) and are briefly summarized here. Upa yoga is a subtype of Hatha yoga and was developed by the Isha Foundation of Inner Sciences. The yoga program was delivered by a certified yoga facilitator from the Isha Foundation. The Upa yoga program consisted of (i) directional movements and neck rotations, (ii) Hatha yoga-based yoga namaskar, (iii) breathing practices or alternate nostril breathing, (iv) mantra meditation consisting of AUM chanting (OM), and (v) breath watching. The intervention was delivered online weekly for 8 weeks. Classes were delivered in sizes of 3–7 participants using the Microsoft Office Team platform for approximately 60 minutes. The participants were also asked to practice at home every day with the support of yoga videos and adherence to home practice was captured using a weekly practice log (see Supplementary File 4, Supplementary Digital Content 4, http://links.lww.com/AJG/C769).

#### Advice-only control group.

Control participants received a 10-minute video including general education on IBS, the mind-gut connection in IBS, and the role of mind-body therapies in the management of IBS. These participants also received a list of IBS-related resources from the Canadian Digestive Health Foundation, a link to an IBS patient support group (www.ibspatient.org), and information about physical activity guidelines from the World Health Organization (see Supplementary File 2, Supplementary Digital Content 2, http://links.lww.com/AJG/C767). The intervention group did not receive these resources.

### Outcome measures

#### Efficacy outcomes.

The intervention and control groups were assessed on efficacy outcomes at baseline and 8 weeks. The primary end point measure was at least a 50-point difference on the IBS Symptom Severity Scale (IBS-SSS) between the groups postintervention (22,24). Scores on the IBS-SSS range from 0 to 500 with higher scores indicating more severe symptoms. Participants can be categorized as having mild (75–175), moderate (176–300), or severe (>300) IBS.

Secondary outcomes (and their measures) include QOL (IBS-QOL) (25), anxiety (Generalized Anxiety Disorder-7) (26), depression (PHQ-9) (27), perceived stress (Perceived Stress Scale) (28), COVID-19–related stress (COVID-19 Stress Scale), fatigue (Modified Fatigue Impact Scale-21) (29), somatic symptoms (Patient Health Questionnaire-15) (30), and self-compassion (Self-Compassion Scale-Short Form) (31).

The Theory of Planned Behavior (TPB) was applied to determine whether the intention to practice yoga at baseline correlated with practice minutes. The TPB is a widely used social-cognitive theory to understand health behaviors in various disease and nondisease populations, including cancer (32), older adults (33), healthy adults (34), cardiac rehabilitation (35), dementia (36), diabetes (37), and rheumatoid arthritis (38). We defined intention based on standardized TPB statements as *doing yoga (behavior) every day (how often) for 30–40 minutes (how long) for the next 8 weeks (length of time)*. Intention to practice yoga was measured from 1 (very unlikely) to 7 (very likely) with “I intend to do yoga daily for 30–40 minutes for the next 8 weeks.”

#### Feasibility outcomes.

*Attrition* was calculated by the percentage of participants who completed all study measures at baseline and 8 weeks. *Adherence* was defined as class attendance of at least 75% (i.e., attendance in 6 of 8 classes) (11) and an attrition rate of less than 30%. *Assessment of harms* was based on any adverse events experienced during the yoga intervention (e.g., physical injury). *Program satisfaction* was evaluated with a survey, including overall rating of the program (poor, ok, good, great, and excellent) and satisfaction with videos and online class instruction on a scale from 1 (strongly disagree) to 7 (strongly agree). Program satisfaction was achieved if at least 70% of participants were satisfied (i.e., rank the program as good, great, or excellent). Participants also indicated whether they would recommend the program to others.

### Sample size

Symptom reduction of at least 50 points on the IBS-SSS is considered clinically meaningful (22). A considerable patient-reported improvement has been determined to be 80 points (22). The sample size (25 per group) was calculated using a mean difference of at least 80 points on the IBS-SSS (⍺ = 0.05, β = 0.80, SD of 103.8) (11). Assuming a 30% attrition rate, we aimed to recruit 33 participants per group.

### Randomization, treatment allocation, and blinding

Participants were randomized after baseline assessment to either the yoga intervention or the advice-only control group. A statistician blinded to the randomization key created a computer-generated REDCap randomized sequence to allocate participants. Participants were aware of the group to which they were allocated. The principal investigator and data analyst remained blinded to the randomization process.

### Data analysis

Participant characteristics and feasibility metrics for both treatment and control groups as well as program adherence for the treatment group only were summarized using descriptive statistics. Fisher exact tests examined baseline differences between groups for categorical variables. Percentages were calculated to determine the proportion of participants in each group who reached clinical significance.

Both intent-to-treat and per-protocol analysis were conducted. Adjusted ANOVA was used to determine the differences between and within groups in the primary and secondary outcomes at 4 weeks and after the intervention. Multiple comparisons (i.e., post hoc) were adjusted using Bonferonni corrections. Multiple multivariate logistic regression was used to examine determinants of response to intervention. Responders were defined as individuals with a reduction of 80 points or more on the IBS-SSS (9). Baseline scores for efficacy outcomes, attendance, and practice minutes were considered for inclusion based on correlation with response. Potential variables with a correlation *P* value greater than 0.20 were included in the regression models to analyze potential determinants of response.

Fisher exact tests were used to determine whether the proportion of individuals falling in each intention category differed between treatment and control groups at baseline and postintervention separately and if any changes in proportions of intentions between baseline and postintervention. In the treatment group alone, regression analysis was used to determine whether intention predicts total yoga practice in minutes and whether there was a relationship between total practice minutes and change (baseline to week 8) in IBS-SSS. All analysis was conducted using RStudio version 1.4.1717 using R version 4.1.1.

This study has been approved by the Conjoint Health Research Ethics Board (REB ID 20-0084).

## RESULTS

### Participant characteristics

**Figure 1.**
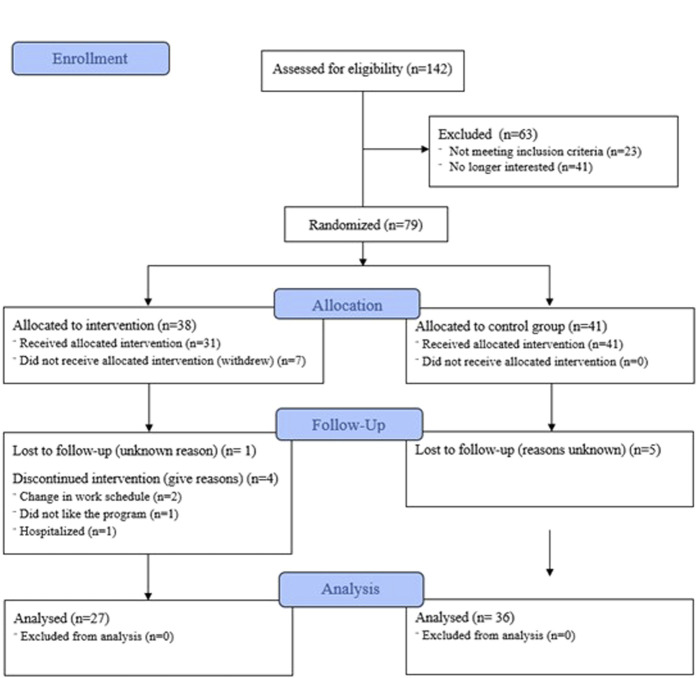
Participant flowchart based on the CONSORT guidelines.

A total of 142 patients expressed interest in participating and 63 were excluded (see Figure 1 for exclusion reasons). The remaining 79 participants were randomized to the yoga group (n = 38) or the control group (n = 41). Patient characteristics are summarized in Table 1. The mean age was 45.4 years (SD = 14.0). Most patients were women (92.4%) and White (81%). The mean IBS diagnosis duration was 11.5 (SD = 10.7) years. There were no baseline differences between groups in sociodemographic variables.

**Table 1.**
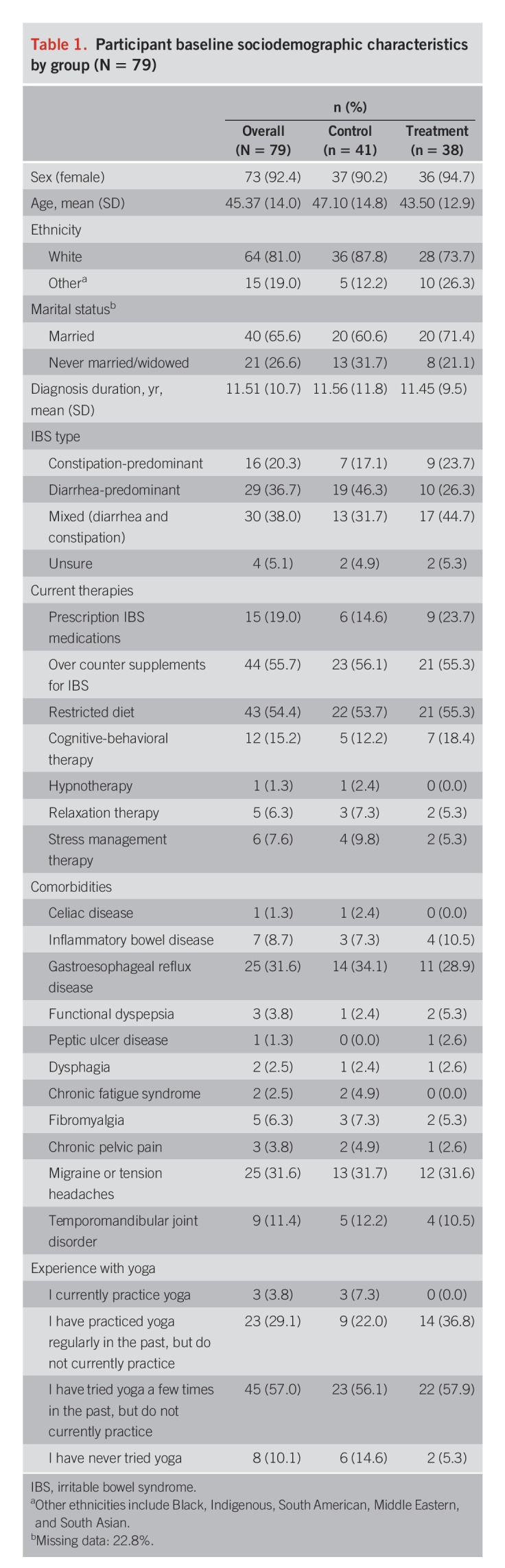
Participant baseline sociodemographic characteristics by group (N = 79)

#### Primary outcome.

The sample mean IBS-SSS was moderate at 245.3 (196.5–317.0, SD = 86.6) points at baseline and 207.9 (117.0–270.0, SD = 100.2) at week 8. The percentage of patients meeting the ≥50-point decrease in IBS-SSS postintervention (8 weeks) was 37% (n = 14) in the yoga group compared with 20% (n = 8) in the control group (*P* = 0.242). The difference between groups in IBS-SSS was 32.1 points, and this difference was not significant (*P* = 0.149) (Table 2). Per-protocol analysis did not reveal different results for the IBS-SSS. In the yoga group, the IBS-SSS score decreased from 255.2 (SD = 90.7) at baseline to 200.5 (SD = 103.9) postintervention (Δ_change_ = 54.7, *P* = 0.028) and from 236.1 (SD = 82.6) at baseline to 213.5 (SD = 98.5) postintervention in the control group (Δ_change_ = 22.6, *P* = 0.277). IBS-SSS improvement in the treatment group was also observed early at 4 weeks from baseline (*P* = 0.006).

**Table 2.**
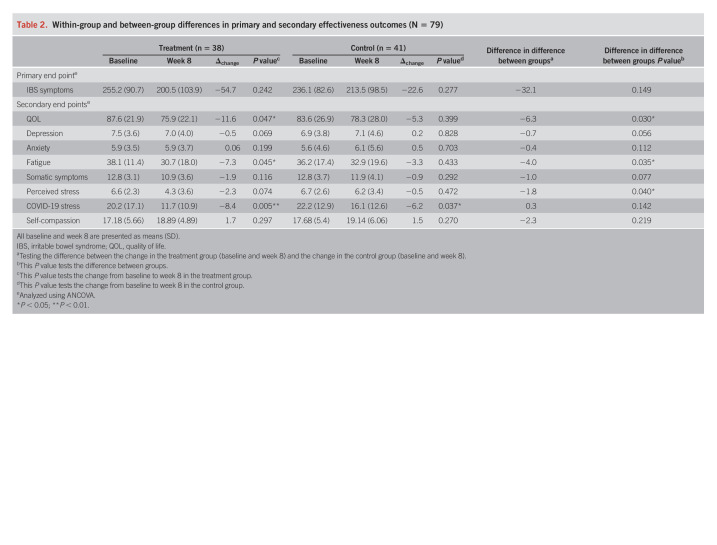
Within-group and between-group differences in primary and secondary effectiveness outcomes (N = 79)

#### Subgroup exploratory analysis of responders.

Twenty-two patients were included in the responder analysis (14 [51.8%] and 8 [22.2%]) (i.e., responders in the treatment and control groups). The difference between the groups was nonsignificant (*P* = 0.143). Responders (n = 22) reported improved IBS symptoms, QOL, perceived stress, and COVID-19 stress. In the treatment group, there were significant improvements in IBS symptoms (Δ = 124.6), QOL (Δ = 19.7), fatigue (Δ = 12.7), somatic symptoms (Δ = 3.4), self-compassion (Δ = 4.4), and COVID-19–related stress (Δ = 14.1). In the control group, responders improved significantly in IBS symptoms (Δ = 132.1) and COVID-19–related stress (Δ = 9.1). The proportion of change (i.e., from baseline to postintervention) in outcome measures for treatment and control groups is shown in Table 3.

**Table 3.**
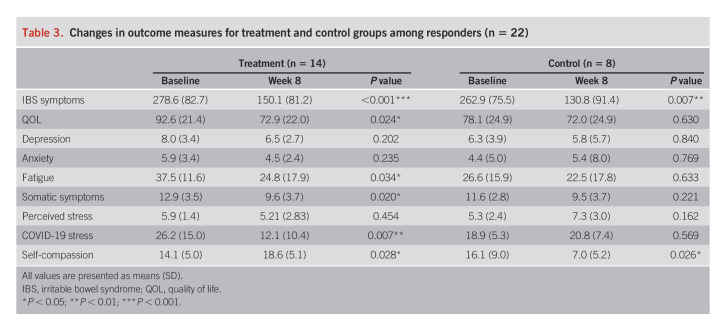
Changes in outcome measures for treatment and control groups among responders (n = 22)

Determinants of response (variables with <0.2 *P* value on their correlations) were perceived stress (*P* = 0.125), self-compassion (*P* = 0.009), COVID-19 stress (*P* = 0.097), and total practice minutes (*P* = 0.005). Perceived stress (odds ratio [OR] = −0.49, 95% CI: 0.25–0.95, *P* = 0.035), self-compassion (OR = 0.68, 95% CI: 0.50–0.93, *P* = 0.016), COVID-19 stress (OR = 1.09, 95% CI: 1.01–1.78, *P* = 0.025), and total practice minutes (OR = 1.003, 95% CI: 1.001–1.004, *P* = 0.007) were predictors of response. The multivariable model, including determinants of response above, suggested that every additional minute a patient practices, they are 1.003 times more likely to be a responder. The mean overall practice minutes in the responder group (for those who received treatment) is 1,213.6. The mean overall practice minutes in the nonresponder group (for those who received treatment) is 594.8, for a difference of 618.8 minutes. Practicing 618.8 minutes more over 8 weeks (or 77.4 minutes per week) resulted in being almost 5 times more likely to be a responder.

#### Secondary outcomes.

Using intent-to-treat analysis, we observed between-group differences postintervention favoring the treatment group for QOL, fatigue, and perceived stress (Table 2). There was a significant improvement in the treatment group in QOL (Δ = −11.6), fatigue (Δ = −7.3), somatic symptoms (Δ = −1.9), perceived stress (Δ = −2.3), and COVID-19–related stress (Δ = −8.4). These benefits were observed as early as week 4 for QOL (*P* = 0.004), fatigue (*P* = 0.003), somatic symptoms (*P* = 0.001), and COVID-19–related stress (*P* < 0.001). In the control group, improvements were seen only in COVID-19–related stress (Δ = −6.2). There were no significant improvements in anxiety, depression, or self-compassion within groups. Although the changes in depression scores are not significant, between-group differences were in favor of yoga (Δ = −0.5) compared with the control group (Δ = 0.2). Anxiety scores were unchanged in both groups. Per-protocol analysis revealed improvement in somatic symptoms (*P* < 0.001) and self-compassion (*P* = 0.003) in the treatment group alone.

### Previous yoga experience and intention to do yoga

Most participants had tried yoga in the past. Intention to do yoga was 6.5 (SD = 0.7) at baseline and 4.0 (SD = 1.8) at 8 weeks for the treatment group and 6.5 (SD = 0.6) at baseline and 5.2 (SD = 1.7) at 8 weeks for the control group. There was a significant change in proportions of intention to do yoga from baseline to postintervention for both groups (treatment *P* < 0.001; control *P* < 0.001). In the treatment group, 97% had high intentions at baseline and 34% postintervention. Low intentions increased from zero percent at baseline to 26% postintervention. In the control group, 100% had high intentions at baseline and 63% at 8 weeks. Low intentions increased from zero percent at baseline to 17% postintervention. These differences were not significant between groups posttreatment (*P* = 0.058). Participants were more likely to indicate neutral and low levels postintervention, and this occurred more frequently for the treatment group. Intention was not significantly associated with yoga practice in minutes (β = 140, t = 1.09, *P* = 0.288) or change in IBS-SSS scores from baseline to postintervention (β = 0.025, t = 0.66, *P* = 0.52).

### Feasibility outcomes

The attrition rate was 20% (29% and 12% in the treatment and control groups, respectively). In the treatment group, 7 of 11 participants were randomized to the intervention but did not start the program, 2 participants withdrew because of changing work schedules, 1 participant was hospitalized for a non–IBS-related concern, and 1 participant did not provide a reason. Average class attendance was 79% (SD = 20%). Treatment participants accumulated an average of 1,220.9 (SD = 513.7) minutes doing yoga. No adverse or safety events were reported. Forty-one percent of participants rated the program as excellent, 30% as great, and 29% as good. All participants strongly agreed that the practice videos and feedback on their practices were helpful and the yoga facilitator was knowledgeable and approachable. Fifty-two percent strongly agreed they would recommend the program to other patients with IBS.

## DISCUSSION

The MY-IBS study is the first to demonstrate the feasibility and safety of an 8-week virtual yoga program combined with the home-based practice for patients with IBS compared with an advice-only control group. The sample had moderate IBS symptom severity at baseline. Significant within-group improvements in IBS symptoms were observed in the treatment group alone at both 4 and 8 weeks after baseline. We did not find significant differences in IBS symptoms between groups postintervention as measured by the IBS-SSS. The response seen in both groups could be explained by the high placebo effect in IBS. Based on a previous meta-analysis of 19 studies examining the placebo response in complementary and alternative medicine trials of IBS, the rate of placebo response was 42.6% (39). The rate is lower (around 30%) when evaluating randomized control trials of licensed IBS drugs for an abdominal pain end point in trials lasting around 6 weeks, with longer trials showing lower placebo rates (40). Future studies evaluating yoga could consider lengthening the duration of the trial to minimize the placebo response rate. Taken together, our data show that virtually delivered yoga is safe and feasible, and effective in reducing IBS symptoms. However, based on the primary end point, the intervention was not superior to an advice-only control group. Similar to our study, in-person yoga trials (4 randomized controlled trials [RCTs]) have yielded either superior results to active comparator arms or improvements in both groups in a variety of outcomes including IBS severity, QOL, anxiety, depression, stress, and somatic symptoms.

We determined predictors of yoga response using a reduction of 80 points on the IBS-SSS. Fifty-two percent of the treatment participants and 22% of the control participants were classified as responders. One other study (11) identified responders using an improvement of 50 points or more on the IBS-SSS as the threshold of clinically significant symptom improvement. This study found 100% of the treatment participants and 22% of controls were responders at week 12. Responders in our study reported improved IBS symptoms, QOL, perceived stress, fatigue, somatic symptoms, and COVID-19–related stress. Our prediction model suggests increased perceived stress is less likely to result in a response, whereas high self-compassion and greater practice time are more likely to result in a response. Although statistically significant, these results are not clinically meaningful and would not change clinical practice. Therefore, future research should continue to explore predictors or response to determine predictive criteria that helps to identify those patients with IBS who are most likely to respond to yoga interventions.

We used a 50-point drop in the IBS-SSS as our primary outcome rather than using the composite outcome mandated by the US Food and Drug Administration for drug trials in IBS (https://www.fda.gov/media/78622/download). The IBS-SSS is a well-validated questionnaire that evaluates the severity and frequency of abdominal pain (22). We did not evaluate changes in stool form or frequency which is a more objective end point because our study included patients with IBS-diarrhea-predominant, IBS-mixed, and IBS-constipation-predominant. Thus, a limitation of our study is that our primary end point is relatively subjective as it evaluates patient-reported pain.

Our study demonstrates beneficial effects of yoga for QOL, fatigue, somatic symptoms, perceived stress, COVID-19–related stress, and self-compassion among patients with IBS. These outcomes were not frequently assessed in the other RCTs. Stress and somatic symptom improvements have been found only in the yoga group in 3 separate RCTs (9,11,12). Fatigue was not assessed in the aforementioned RCTs, and based on a meta-analysis examining fatigue prevalence in IBS, the search did not yield any interventional studies for comparison (41). However, the literature on other chronic diseases such as cancer (42) and multiple sclerosis (43) suggests yoga interventions are effective at managing fatigue. Although not primary outcomes, these results are of interest given the high proportion of patients with IBS who experience stress, fatigue, and low QOL. Until high-quality studies examine the effects of yoga on these measures as primary outcomes, clinicians may consider recommending yoga in these specific scenarios.

We did not observe any within-group or between-group differences for anxiety and depression, especially considering the evidence base for yoga for each condition (44,45). The lack of significance may be driven both by the low baseline levels of anxiety and depression and a sample size powered to detect changes in IBS symptoms (but not depression or anxiety). Similar to this study, other IBS RCTs reported mixed findings, with 2 studies finding improvements in anxiety (12) and depression (9,12) in the yoga group and another study not finding any significant between group differences in anxiety, although, within-group differences were observed for both groups (10). By contrast, another RCT found both within-group and between-group differences (11). The differences in yoga interventions and comparator groups make it challenging to compare our findings with other studies as some of these studies did not report baseline anxiety and depression.

The yoga intervention in this study was feasible for adherence (79%), attrition rate (20%), and high program satisfaction. Adherence was reported in 3 of the previously described RCTs, varying from 62% to 90% (9,11,12). The attrition rate is also comparable to the other RCTs varying from 5% to 24%. Safety was also demonstrated without any adverse events. We also explored participants' intention to practice yoga over the next 8 weeks. Intention to practice yoga was higher at baseline in the treatment group, but there was a significant decrease in proportion in intentions for both groups. A decrease of 63% in intention from baseline to postintervention among the treatment group is unexpected, especially considering the demonstrated feasibility and high satisfaction with the program. Although intention to practice yoga diminished with time through the study period, this did not affect practice time, suggesting that factors outside of intention may be important to explore in future studies. We suggest future studies include a midstudy check-in with participants to measure intention and conduct poststudy interviews to explore participant's experiences in the intervention, including any major changes in intention.

### Strengths and limitations

This study is the first to evaluate the efficacy and feasibility of a virtual yoga intervention. The yoga intervention was developed based on IBS mechanistic rationale supported by an international yoga foundation and delivered by an experienced yoga instructor and at-home practice was supported by videos. We recruited patients from across Canada, using heterogeneous recruitment methods including gastroenterologist offices, primary care physician clinics, and social media. These results may not be generalizable as our sample is largely composed of educated White women with a high family income. Our data are also limited by the lack of data capture on the frequency of yoga practice, if any, in the control group, and lack of information as to the time that had passed between previous yoga practice and the current study. Our sample did not include patients with severe depression. Finally, 7 of 11 treatment participants did not start the intervention.

Future studies should consider including objective measures of autonomic function testing (e.g., electrocardiogram), sympathetic reactivity tests (e.g., mental arithmetic test), and parasympathetic reactivity tests (e.g., heart rate variability in deep breathing) to determine physiological mechanisms of yoga response. Future studies with a larger sample size should also investigate the effects of yoga on IBS subgroups (IBS-diarrhea-predominant, constipation-predominant, or mixed) individually and the long-term effects of yoga.

Our study results suggest that virtually delivered yoga is safe and feasible. Yoga improved IBS symptoms and a breadth of other psychological and physiological outcomes that are understudied but frequently affect patients with IBS. The intervention was also found to be safe without any adverse events. The virtual delivery of yoga represents an opportunity to increase access to effective management therapies for patients with IBS. Combining the convenience and flexibility of virtual programs and the social benefits of in-person interactions into hybrid programming may improve program efficacy, intervention adherence, and patient outcomes.

## CONFLICTS OF INTEREST

**Guarantor of the article:** Maitreyi Raman, MD, MSc.

**Specific author contributions:** A.D. was involved in all aspects of study design, recruitment, data collection. D.A.M., J.K.V., Y.N., V.R., and M.R. assisted with study design respective to their expertise. J.H.S. led the statistical analysis. All authors reviewed the manuscript for study design and provided critical insight into manuscript content and approved the final version for submission.

**Financial support:** This research received no specific grant from any funding agency in the public, commercial, and not-for-profit sectors. D.A.M. receives partial salary support through the Arthur J.E. Child Chair.

**Potential competing interests:** None to report.

**Trial registration number:** NCT04302623.

**Study Highlights**

**WHAT IS KNOWN**

✓ In-person yoga interventions are effective in managing irritable bowel syndrome (IBS) symptoms.
✓ A virtually delivered yoga intervention has not been tested in IBS.

**WHAT IS NEW HERE**

✓ Virtually delivered yoga improved IBS symptoms in the treatment group, but not the control.
✓ Quality of life, fatigue, perceived stress, and COVID-19–related stress also improved.
✓ Virtually delivered yoga program was also safe and feasible.

## Supplementary Material
