# Supplementary material for: Meditation and Yoga for Irritable Bowel Syndrome: A Randomized Clinical Trial
Source: Am J Gastroenterol. 2022 Oct 11;118(2):329–37. doi: 10.14309/ajg.0000000000002052 (PMC9889201; doi:10.14309/ajg.0000000000002052)
Supplement: Supplementary file 1 [file acg-118-329-s001.pdf]

UNIVERSITY OF CALGARY

IMAGINE

Ascend

Meditation and Yoga for Irritable Bowel Syndrome (MY-IBS) Study

WEEKLY PRACTICE LOG

Please DOWNLOAD this form on your computer and SAVE it after you fill it every day.

Week # Select one

Participant Id

Week Start Date

Check the box for each practice you completed and record the total daily minutes of your practice. Skip the rows for the practices you have not yet learned.

Yoga practices completed	Day 1	Day 2	Day 3	Day 4	Day 5	Day 6	Day 7	Week Total
Directional Movement of the Arms								0
Neck Practices								0
Yoga Namaskar								0
Alternate Nostril Breathing								0
Mantra Meditation								0
Breath Watching								0
Total daily minutes spent doing Yoga								0

Click the RESET button to clear and enter values for a new week RESET

If you have finished entering all the values for this week, please go to the next page.

The following 7 questions are **OPTIONAL** and can be completed **ONLY** after you have finished entering all your practices for this week.

These questions will help to record and monitor the severity of your IBS symptoms on a weekly basis. It is to be expected that your symptoms might vary over time, so please try and answer the questions based on how you currently feel.

If you answer all of the questions, you will be able to see your IBS symptom severity score for this week

1. Do you currently suffer from abdominal pain? ○ **YES** ○ **NO**

2. If Yes, how severe is your abdominal pain?

○ No pain ○ Not very severe ○ Quite severe ○ Severe ○ Very severe

3. Please enter the number of days that you get pain every **10** days - (*For example, if you enter 4, it means that you get pain 4 out of 10 days. If you get pain every day, enter 10).*

4. Do you currently suffer from abdominal distension (bloating, swollen or tight stomach)?[*People who menstruate, please ignore distension related to your periods].* ○ **YES** ○ **NO**

5. If Yes, how severe is your abdominal distension/tightness?

○ No distension ○ Not very severe ○ Quite severe ○ Severe ○ Very severe

6. How satisfied are you with your bowel movements?

○ Very satisfied ○ Quite satisfied ○ Neutral ○ Quite dissatisfied ○ Very dissatisfied

7. How much is your IBS affecting or interfering with your life in general?

○ Not at all ○ Not much ○ Somewhat ○ Quite a lot ○ Completely

Your IBS Severity Score **0**

What your score means

75-175: Mild
175-300: Moderate
>300: Severe

*Please click on **SUBMIT** below to send us your weekly Practice log.*

SUBMIT Practice Log

To send us the paper form, please scan and email it to vidya.rajagopalan@ucalgary.ca
