# Supplementary material for: Meditation and Yoga for Irritable Bowel Syndrome: A Randomized Clinical Trial
Source: Am J Gastroenterol. 2022 Oct 11;118(2):329–37. doi: 10.14309/ajg.0000000000002052 (PMC9889201; doi:10.14309/ajg.0000000000002052)
Supplement: Supplementary file 2 [file acg-118-329-s002.pdf]

UNIVERSITY OF CALGARY

RESOURCES FOR CONTROL PARTICIPANTS

Here are a few resources on IBS you may find helpful.

General Information on IBS:

https://cdhf.ca/digestive-disorders/irritable-bowel-syndrome-ibs/
https://aboutibs.org/

IBS Patient Support Group:

https://www.ibspatient.org/

Physical Activity Information:

https://www.who.int/news-room/fact-sheets/detail/physical-activity

We hope you found this information helpful.

Should have any questions about this study, please contact us.

Adrijana D'Silva
Study Coordinator
adrijana.dsilva@ucalgary.ca
