# Supplementary material for: Meditation and Yoga for Irritable Bowel Syndrome: A Randomized Clinical Trial
Source: Am J Gastroenterol. 2022 Oct 11;118(2):329–37. doi: 10.14309/ajg.0000000000002052 (PMC9889201; doi:10.14309/ajg.0000000000002052)
Supplement: Supplementary file 3 [file acg-118-329-s003.docx]

**COVID-19 Stress Scales**

**The following asks about various kinds of worries that you might have experienced over the past seven days. In the following statements, we refer to COVID-19 as "the virus".**

| 0 | 1 | 2 | 3 | 4 |
| --- | --- | --- | --- | --- |
| Not at all | Slightly | Moderately | Very | Extremely |

| 1. I am worried about catching the virus |
| --- |
| 1. I am worried that basic hygiene (e.g., handwashing) is not enough to keep me safe from the virus |
| 1. I am worried that our healthcare system is unable to keep me safe from the virus |
| 1. I am worried that I can’t keep my family safe from the virus |
| 1. I am worried that our healthcare system won’t be able to protect my loved ones |
| 1. I am worried that social distancing is not enough to keep me safe from the virus |
| 1. I am worried about grocery stores running out of food |
| 1. I am worried about grocery stores running out of cold or flu remedies |
| 1. I am worried about pharmacies running out of prescription medicines |
| 1. I am worried about grocery stores running out of water |
| 1. I am worried about grocery stores running out of cleaning or disinfectant supplies |
| 1. I am worried that grocery stores will close down |
| 1. I am worried that foreigners are spreading the virus in my country |
| 1. If I met a person from a foreign country, I’d be worried that they might have the virus |
| 1. I am worried about coming into contact with foreigners because they might have the virus |
| 1. I am worried that foreigners are spreading the virus because they’re not as clean as we are |
| 1. If I went to a restaurant that specialized in foreign foods, I’d be worried about catching the virus |
| 1. If I was in an elevator with a group of foreigners, I’d be worried that they’re infected with the virus |
| 1. I am worried that people around me will infect me with the virus |
| 1. I am worried that if I touched something in a public space (e.g., handrail, door handle), I would catch the virus |
| 1. I am worried that if someone coughed or sneezed near me, I would catch the virus |
| 1. I am worried that I might catch the virus from handling money or using a debit machine |
| 1. I am worried about taking change in cash transactions |
| 1. I am worried that my mail has been contaminated by mail handlers |

**In the following statements, we refer to COVID-19 as "the virus". Please read each statement and indicate how frequently each problem has been for you during the past seven days.**

| 0 | 1 | 2 | 3 | 4 |
| --- | --- | --- | --- | --- |
| Never | Rarely | Sometimes | Often | Almost always |

| 1. I had trouble sleeping because I worried about the virus |
| --- |
| 1. I had bad dreams about the virus |
| 1. I thought about the virus when I didn’t mean to |
| 1. Disturbing mental images about the virus popped into my mind against my will |
| 1. I had trouble concentrating because I kept thinking about the virus |
| 1. Reminders of the virus caused me to have physical reactions, such as sweating or a pounding heart |

**The following items ask about checking behaviours. During the past seven days, how much have you checked the following because of concerns about COVID-19?**

| 0 | 1 | 2 | 3 | 4 |
| --- | --- | --- | --- | --- |
| Never | Rarely | Sometimes | Often | Almost always |

| 1. Social media posts concerning COVID-19 |
| --- |
| 1. YouTube videos about COVID-19 |
| 1. Seeking reassurance from friends or family about COVID-19 |
| 1. Checking your own body for signs of infection (e.g., taking your temperature) |
| 1. Asking health professionals (e.g., doctors or pharmacists) for advice about COVID-19 |
| 1. Searched the Internet for treatments for COVID-19 |
